# Comparison of Optimal and Excess Fresh Gas Flow for Pre-intubation Oxygenation: A Randomized, Participant-Blind, Non-inferiority Study

**DOI:** 10.7759/cureus.100807

**Published:** 2026-01-05

**Authors:** Girish C Pathak, Habib Md R Karim, Subrata K Singha, Jitendra V Kalbande, Chinmaya K Panda

**Affiliations:** 1 Anaesthesiology and Critical Care, All India Institute of Medical Sciences, Raipur, Raipur, IND; 2 Anaesthesiology, Critical Care, and Pain Medicine, All India Institute of Medical Sciences, Guwahati, Guwahati, IND; 3 Anaesthesiology and Critical Care, All India Institute of Medical Sciences, Nagpur, Nagpur, IND

**Keywords:** closed circuit anesthesia, denitrogenating, fresh gas flow, noninvasive ventilation for preoxygenation, pre-intubation oxygenation, pre-oxygenation efficacy

## Abstract

Background: The effect of different fresh gas flow (FGF) on achieving the target expired oxygenation (FeO_2_) has been evaluated mainly with manual bag-mask ventilation, which provides variable minute ventilation (MV). The effect of machine-delivered consistent MV has yet to be well established. The present study evaluated the efficacy of pre-intubation oxygenation using optimal and excess FGF.

Methods: One hundred adult patients aged 18-60 years and with American Society of Anesthesiologists' physical status I-III were randomized to receive optimal FGF (=MV+500 mL) or excess FGF (i.e., +50%) for pre-intubation oxygenation targeted to FeO_2_ of 85% and 90% or ventilated to maximum for five minutes. Concurrent fixed dial settings of volatile agents were used. Ventilation was done using an anesthesia machine in a pressure support (PS)-based technique. MV, fraction of inspired oxygen (FiO_2_), and FeO_2 _were collected and compared at different timepoints.

Results: Both optimal and excess FGF groups showed similar efficacy in achieving FeO_2_. The mean FeO_2_ in the optimal and excess group at three minutes was 84.52 ± 3.38% versus 84.94 ± 3.73%; p = 0.368, while the same at five minutes was 86.02 ± 3.12% versus 85.98 ± 3.57%; p = 0.615. The mean time required to achieve an FeO_2_ of 85% was 160.16 ± 67.15 seconds in the optimal FGF group and 147.81 ± 55.50 seconds in the excess FGF group (p = 0.335), with a mean difference of 12.35 seconds (8.36%).

Conclusion: Pre-intubation oxygenation using the optimal FGF is non-inferior to excess FGF, and is as adequate as using excess FGF to achieve FeO_2_ levels exceeding 85% during machine-delivered PS-based ventilation. However, it was noted that three minutes was insufficient to achieve the target FeO_2_ 90% using a closed circuit system and concurrent volatile agents.

## Introduction

Preoxygenation refers to replacing lung nitrogen with oxygen, ensuring sufficient oxygen reserves before airway management and interventions [1,2]. Preoxygenation primarily replaces nitrogen in the functional residual capacity (FRC) of the lungs with oxygen, and recently, machine-delivered noninvasive ventilation techniques have been used with success [3,4]. FRC is the reserve that delays the onset of arterial oxyhemoglobin desaturation during apnea, prolonging the apnea time, defined as the length of time from cessation of breathing or ventilation until the onset of significant arterial desaturation, typically, an oxyhemoglobin saturation <90% [5]. The risk increases tremendously if an unexpected difficult intubation occurs [6]. Preoxygenation is considered the new minimum standard of care during induction of anesthesia, and different societies of on guidance for the difficult airway management emphasize the "face mask preoxygenation before initiating management of the difficult airway” [7,8]. The same risk persists for an unanticipated difficult airway [8]. Therefore, preoxygenation is preferable and ought to be carried out before general anesthesia (GA) induction [1].

However, pre-intubation oxygenation as a denitrogenating technique is also used, especially when difficult mask ventilation is not anticipated, even in emergency settings [3,4]. The key to achieving maximum alveolar nitrogen washout is delivering 100% O_2_ along with optimal alveolar ventilation without re-breathing. In a subject with normal lung function, the O_2_ wash-in and the nitrogen washout are exponential functions governed by the exponential curves' time constant (τ). After four τ, the O_2_ concentration of FRC will be increased by about 98% of its original value [9]. If the breathing is delivered using a machine, it provides uniform minute ventilation (MV), and the pre-intubation oxygenation becomes more efficacious compared to manual bag and mask ventilation (BMV) [10]. The modern machine employing closed-circuit technology and lower flow will result in rebreathing [11]. As a result, the fresh gas flow (FGF) is typically maintained at a level equal to or greater than the MV. Over the years, studies have been done on the FGF, tidal volume (Vt), and breathing pattern with varied results [9]. Most of these studies have used manual BMV, which has an inconsistent delivery of MV [10]. There are limited data and studies on increasing FGF and the rapidity of oxygenation, while maintaining a fixed machine-delivered Vt breathing technique for preoxygenation in patients scheduled for laryngoscopy with endotracheal intubation (LETI), which makes the actual impact of excess FGF unclear.

The present study was designed to compare the effectiveness of adequate oxygenation using the tidal volume breathing (TVB) technique with pressure support ventilation with positive end-expiratory pressure (PSV + PEEP) on a circle absorber system with variable FGF. The study compared two approaches: one in which the FGF was kept optimal, equal to the MV, and another in which the FGF was set high, at 150% of the optimal MV.

## Materials and methods

Study design

The present study was a prospective, randomized, parallel-arm, patient-blinded, non-inferiority study conducted in the operating theatre of an academic institute in India. The study protocol was reviewed by the thesis review committee at the institute and subsequently by the institute's ethics committee, and approval was obtained (No. 2569/IEC-AIIMSRPR/2022 dated 02 November 2022). The study was initiated after registration in the Clinical Trial Registry of India (CTRI/2022/11/047769, dated 29 November 2022), and recruitment and data collection were conducted between January 2023 and July 2024. Informed and written consent was obtained from each study participant. The study adhered to the Good Clinical Practice guidelines and the Declaration of Helsinki, and the results were reported in accordance with the Consolidated Standards of Reporting Trials (CONSORT) guidelines [12].

Operational definitions

In the present study, pre-oxygenation refers to pre-intubation oxygenation, which involves oxygenation using 100% O_2_ via a bag and mask connected to a closed-circuit system, and ventilation through the machine before LETI.

Participants

Male and female individuals aged 18 to 60 with a body mass index (BMI) of 18.5-29.9 kg/m^2^ and a room air peripheral oxyhemoglobin saturation (SpO_2_) of more than 96% undergoing elective surgeries under GA with LETI were enrolled in the study. Exclusion criteria included patient refusal, respiratory diseases such as chronic obstructive airway disease, asthma, and respiratory failure; cardiac diseases like heart failure; patients with anticipated difficult intubation; inability to obtain a proper-fit mask; and moderate to severe obstructive sleep apnoea (OSA). OSA categorization was performed based on the screening results, and a sleep study was not conducted for confirmation. All patients underwent STOP-BANG (Snoring, Tiredness, Observed apnea, high blood Pressure, Body mass index, Age, Neck circumference, and Gender) screening in the preoperative evaluation clinic, and those with probable OSA were excluded [13].

Randomization, allocation, and blinding

Participants were randomly allocated to either of the two groups (Group A: Optimal FGF - preoxygenation was done with optimal FGF, and Group B: Excess FGF - preoxygenation was done with 1.5xFGF based on software-generated random codes). Block randomization with fixed-size blocks was performed using computer-generated block random codes and groups. Randomization blocks and sequences were generated online using Sealed Envelope (Sealed Envelope Ltd., London, United Kingdom) with a seed value of 245247034471792 and unique random codes for each participant [14]. One hundred twenty (initially 100 and then another 20) random numbers were generated and divided into 30 (25 + 5) blocks, each containing four participants. The group allocation was based on his/her recruitment sequence and the random group division within the block. Allocation concealment was maintained by keeping the random blocks and codes central with the thesis guide of the project. The group allocation was disclosed to the primary investigator only after the patient was inside the operating room, and a plan for delayed sequence or rapid sequence intubation was made by the designated consultant of that operating room.

Intervention technique

In the operating room, patients were positioned with their head-end and neck extended so that the sternal notch and the external auditory meatus remained in the same horizontal plane. Standard American Society of Anesthesiologists (ASA) monitoring was initiated, and the same pulse oximetry brand was employed for all patients. Heavy sedation as a premedication was not provided to any patients, and GA induction was standardized. The patient was induced using a titrated sleeping dose of propofol and fentanyl 2 mcg/Kg. A muscle relaxant (vecuronium 0.1 mg/Kg) was administered only after ensuring assisted ventilation. The case was excluded if the patient was found difficult to ventilate. The patient was then shifted to ventilation from the machine. All groups were ventilated noninvasively using PSV + PEEP of 5 cm H_2_O using the Draeger Primus Machine (Drägerwerk AG & Co. KGaA, Lübeck, Germany) and circuit with dial isoflurane setting of 1% by using an appropriate size tight-fitting mask. The FGF was set as per the calculation and group allocation. Group A (optimal FGF) was defined as optimal MV + leaks of the circuit as determined during pre-anesthesia check-up of the machine + 500mL as a margin of safety, and the final value was rounded up to multiples of 500 mL multiplication on the higher side. Group B (excess FGF) was defined as 150% of optimal MV + leaks of the circuit as determined during pre-anesthesia check-up of the machine, and was rounded up to multiples of 500 mL multiplication on the higher side. The optimal MV was calculated for each patient as the Vt of 8 mL/Kg predicted body weight (PBW) @ 15 breaths per minute. PS was started at 12 cmH_2_O and titrated to make it near 8 mL/kg predicted within the next few breaths.

After five minutes, a 0.5 mg/kg bolus dose of propofol was administered, and the patient was intubated using the direct laryngoscopy technique. Capnography and auscultation confirmed the correct position of the endotracheal tube. After that, standard management was followed for both ventilation and perioperative management.

Outcome variables

Preoperative patient demographics, SpO_2_, and basic hemodynamic parameters were noted. MV, Vt, SpO_2_, respiratory rate, FiO_2_, FeO_2_, end-tidal carbon dioxide (EtCO_2_), and alarms were noted each minute for five minutes during the intervention phase in both groups. Also, this phase noted the time taken for pre-oxygenation till FeO_2_ of 85% and 90%.

Sample size

The current study's sample size was calculated using the data from the pilot study conducted for pre-intubation oxygenation using a machine PS + PEEP [10]. The study showed the mean + standard deviation (SD) time required to reach FeO_2_ of 85% in 189.166±56.69 seconds [10]. As we also use the same ventilation technique, we expected a similar SD, i.e., 57 seconds, in our cohort. Further, we took a 10% allowable difference in mean, i.e., (d=μT-μC) = 10% of 189 = 18.9, and set the margin at 20% of SD (which is nearly 6% of the mean), i.e., 20% of 57 = 11.4. The final sample was calculated for a continuous outcome for a 1:1 allocation, absolute precision, or type I error rate (α) of 5% and power (1-β) of 80%, which gave a sample of 44 participants per group; a total of 88. A 12% drop-out margin was added to offset the post-recruitment exclusion, and the final sample is rounded up to 50 participants per group. The sample size was calculated using the Cleveland Clinic Risk Calculator Library online sample size calculator (Cleveland Clinic, Cleveland, Ohio, USA) for a non-inferiority trial [15].

Data management and statistical analysis

The master chart was prepared by entering the data from the case record form into Excel (Microsoft Corp., Redmond, WA, USA). Categorical variables were presented on a number and percentage scale. Continuous data were presented as mean ± SD. Group comparison was performed using unpaired Student t-tests, Fisher’s exact test, chi-squared test, and Wilcoxon-Mann-Whitney U Test as considered appropriate based on the data distribution normality. A two-tailed p-value less than 0.05 was considered statistically significant. IBM SPSS Statistics for Windows, Version 23 (Released 2016; IBM Corp., Armonk, New York, United States) was used for statistical analysis.

## Results

**Figure 1:**
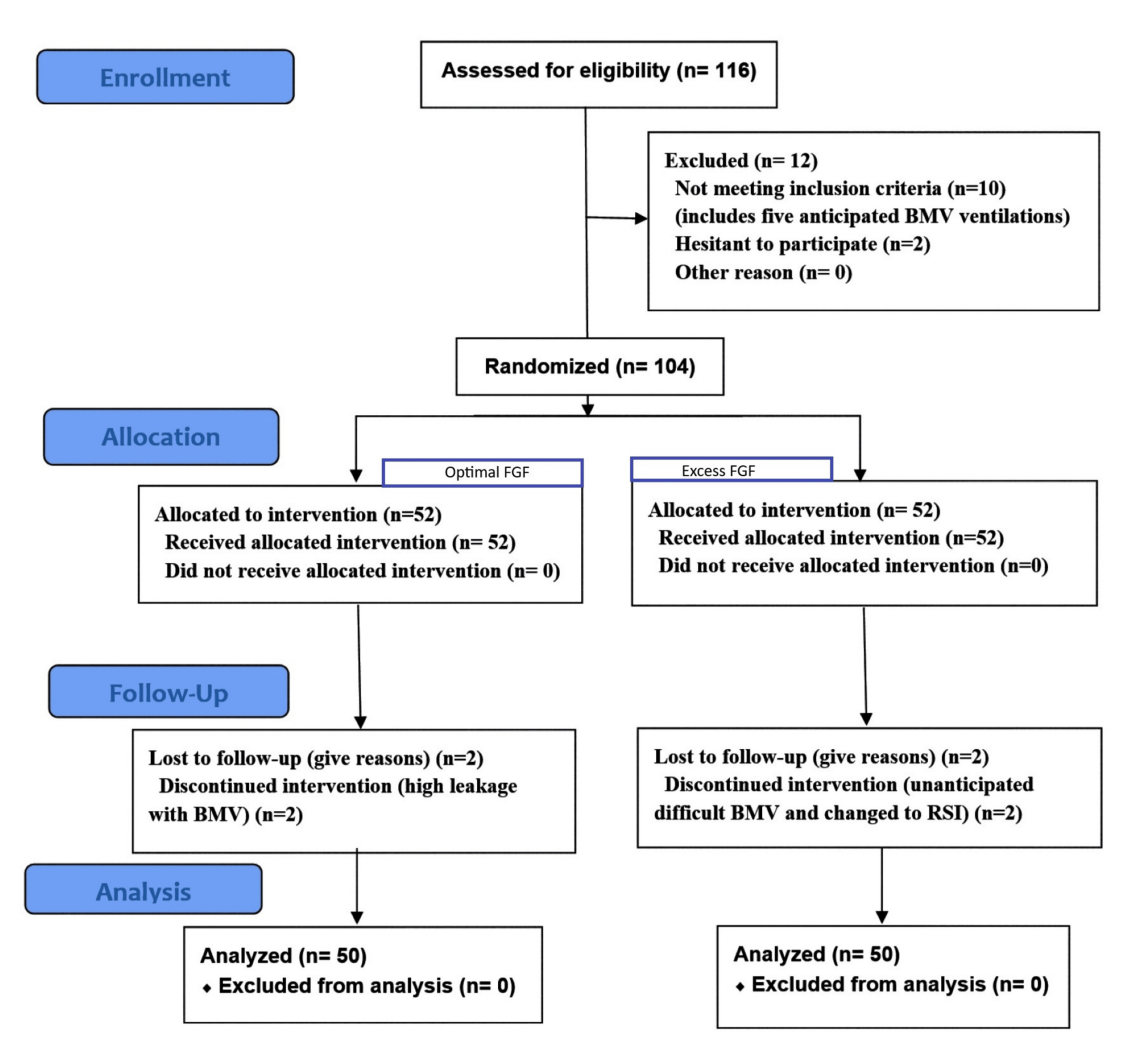
Consolidated Standards of Reporting Trials (CONSORT) flow diagram of the study participants. BMV: bag-mask ventilation, FGF: fresh gas flow, RSI: rapid sequence induction

This study included final data from 100 participants, 50 patients in each group. The CONSORT flow diagram for participants is presented in Figure 1.

The study did not require any follow-up, and data were complete for all cases. However, the time to reach the target FeO_2_ was calculated only from the participants who reached the targets at specific time points. The demographic variables of the two groups were similar in terms of age and actual weight, but in terms of gender, height, BMI, and PBW, they were statistically different. Both groups had similar baseline clinical parameters, such as ASA-PS, smoking, heart rate, respiratory rate, and SpO_2_ (Table 1).

**Table 1:** Clinico-demographic variables and anesthesia system-related data and their comparison. A two-tailed p < 0.05 was considered statistically significant. ^@^ t-test; ^#^ Chi-squared test; ^$^ Wilcoxon-Mann-Whitney U test; * Fisher’s exact test ASA-PS: American Society of Anesthesiologists-Physical Status, BMI: body mass index, FGF: fresh gas flow, NA: not applicable, NICE: National Institute for Health and Care Excellence, SpO_2_: peripheral oxyhemoglobin saturation

Parameters	Optimal FGF (n = 50)	Excess FGF (n = 50)	χ^2^/t/W value	p-value
Age (years)	34.38 ± 11.71	37.78 ± 12.68	t = -1.393	0.167^@^
Age group distribution				0.594^#^
18-30 years	22 (44.0%)	18 (36.0%)	χ^2^ = 0.375	
31-40 years	11 (22.0%)	9 (18.0%)	χ^2^ = 0.062	
41-50 years	12 (24.0%)	14 (28.0%)	χ^2^ = 0.052	
51-60 years	5 (10.0%)	9 (18.0%)	χ^2^ = 0.748	
Male	27 (54.0%)	16 (32.0%)	χ^2^ = 4.080	0.026^#^
Female	23 (46.0%)	34 (68.0%)		
Height (cm)	161.96 ± 8.30	156.82 ± 7.92	t = 3.165	0.002^@^
Actual weight (Kg)	57.48 ± 8.45	59.14 ± 8.41	t = -0.984	0.328^@^
Predicted body weight (Kg)	57.31 ± 7.57	51.10 ± 8.60	W = 1797.5	<0.001^$^
Actual BMI (Kg/m^2^)	21.97 ± 2.67	23.98 ± 3.22	W = 792.000	0.002^$^
BMI				0.013^#^
18.5-24.9 Kg/m^2^	42 (84.0%)	31 (62.0%)	χ^2^ = 5.074	
25-29.9 Kg/m^2^	8 (16.0%)	19 (38.0%)		
ASA-PS				0.289*
I	32 (64.0%)	25 (50.0%)	NA	
II	13 (26.0%)	21 (42.0%)	NA	
III	5 (10.0%)	4 (8.0%)	NA	
NICE Surgical Grade				0.078*
Grade 1	0 (0.0%)	0 (0.0%)	NA	
Grade 2	25 (50.0%)	19 (38.0%)	NA	
Grade 3	20 (40.0%)	30 (60.0%)	NA	
Grade 4	5 (10.0%)	1 (2.0%)	NA	
Smoking history (yes)	9 (18.0%)	11 (22.0%)	χ^2^ = 0.062	0.617^#^
Heart rate (/minute)	81.30 ± 15.34	83.48 ± 15.33	W = 1147.5	0.481^$^
Respiratory rate (/minute)	14.88 ± 1.24	14.90 ± 1.57	W = 1231.0	0.888^$^
SpO_2_ (%)	99.62 ± 0.70	99.60 ± 0.81	W = 1207.0	0.699^$^
Anesthesia workstation used				0.495*
Drager	50 (100.0%)	48 (96.0%)	NA	
Topnotch	0 (0.0%)	2 (4.0%)		
Breathing circuit				0.495*
Limb-O circuit	0 (0.0%)	2 (4.0%)	NA	
Two-limb conventional circuit	50 (100.0%)	48 (96.0%)		

**Figure 2:**
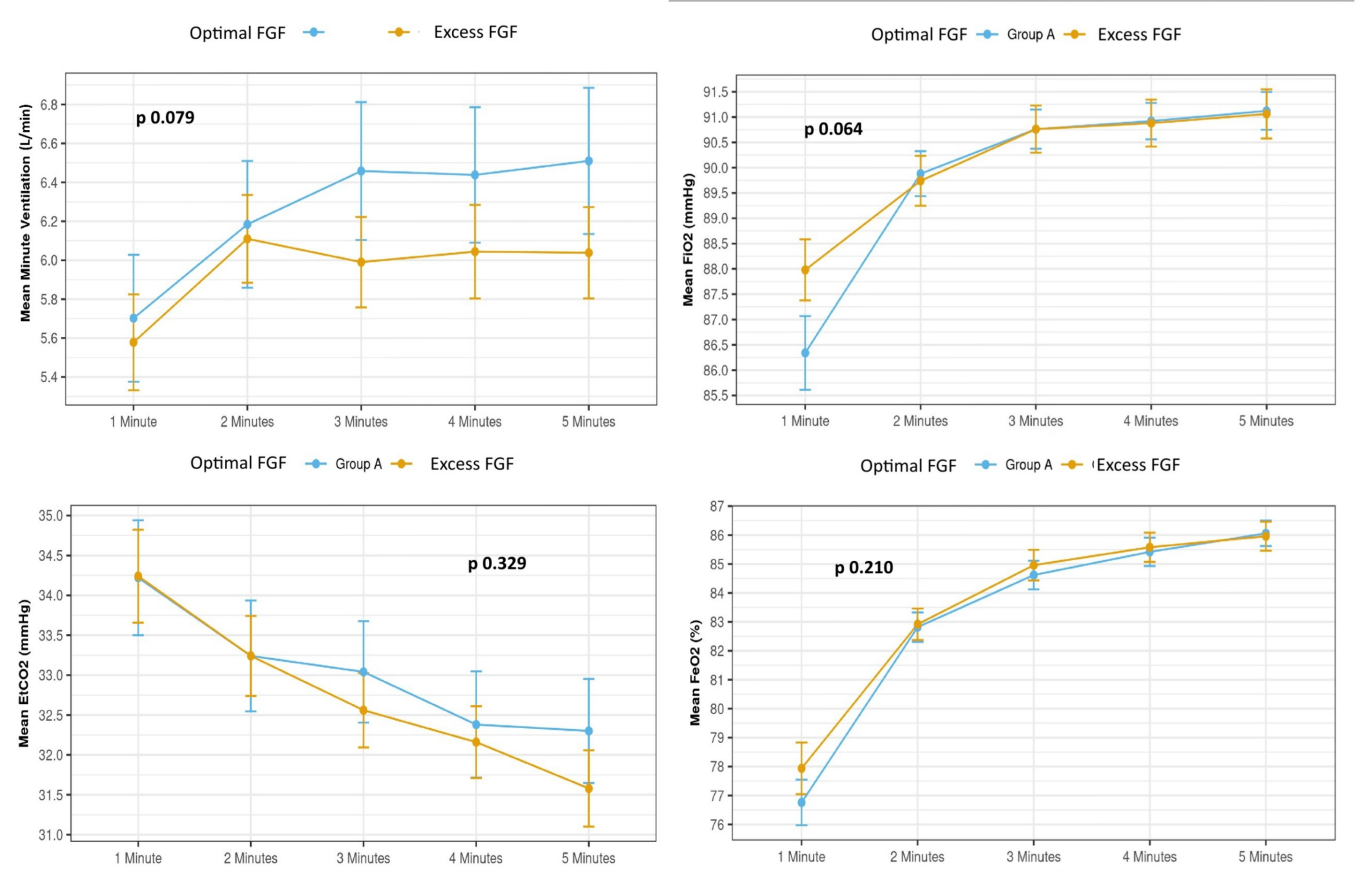
Line graphs showing the mean and standard deviation trends of MV, FiO₂, EtCO₂, and FeO₂ over five minutes of pre-intubation oxygenation in the optimal and excess FGF groups. Overall p-values were derived using the Wilcoxon-Mann-Whitney U test. EtCO_2_: end-tidal carbon dioxide, FGF: fresh gas flow, FiO_2_: fraction of inspired oxygen, FeO_2_: fraction of expired oxygen, MV: minute ventilation

There were no statistically significant differences between groups for mean MV (p = 0.079), mean FiO_2_ (p = 0.064), or mean EtCO_2_ (p = 0.329), over the five-minute observation period. The mean and SD of the data trend and comparison are presented in Figure 2.

The comparison (%) of FeO_2_ reached over time at three and five minutes in both groups was comparable. In optimal FGF, the mean (SD) FeO_2_ reached was 84.52 (3.38)% at three minutes and 86.02 (3.12)% at five minutes, while the same for the excessive FGF were 84.94 (3.73)% and 85.98 (3.57)%, respectively. The mean FeO_2_ achieved over the five minutes was similar (p = 0.210).

**Figure 3:**
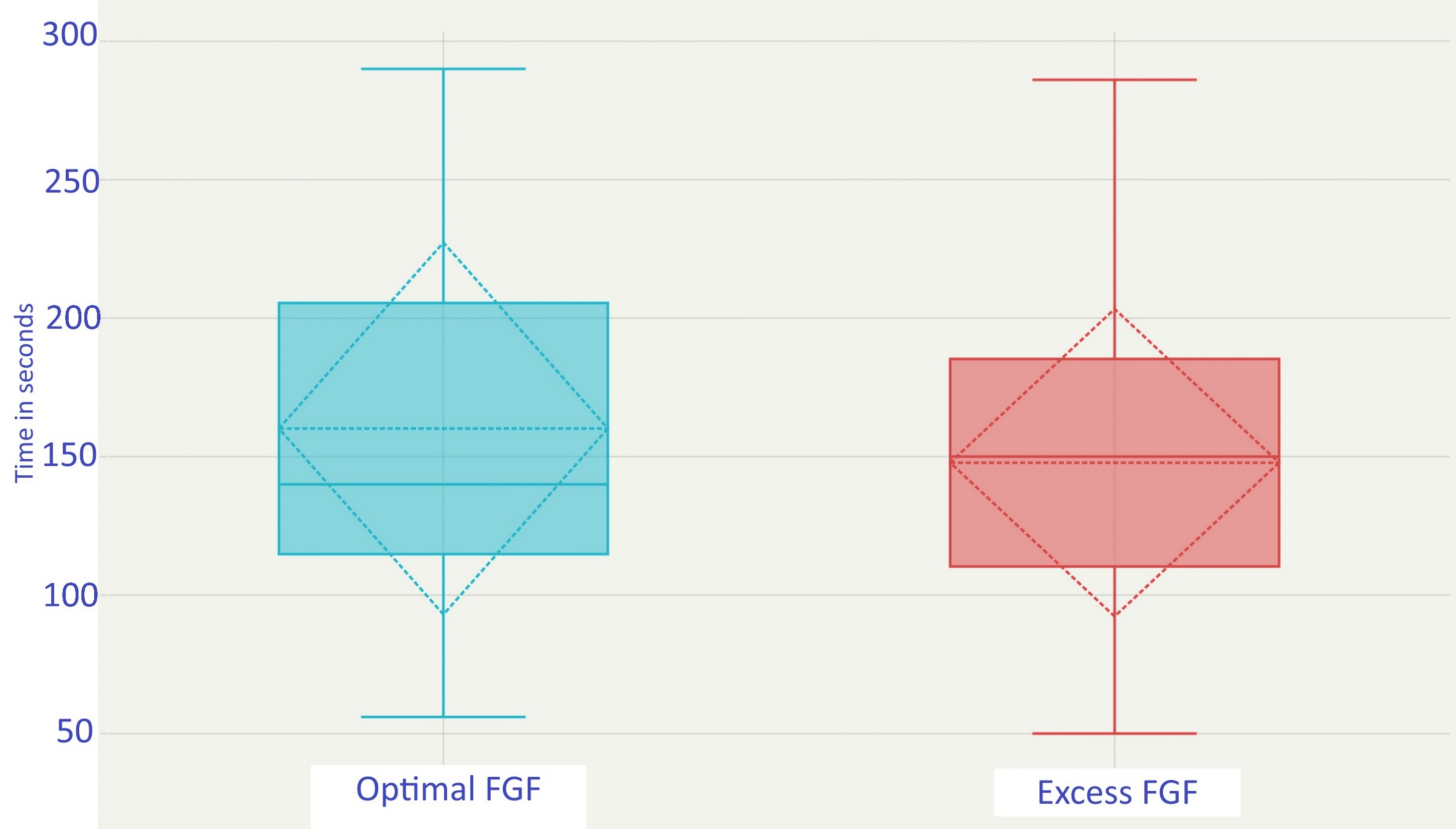
Box-and-whisker plot illustrating the time required to achieve an FeO₂ of 85%. The box represents the interquartile range, the solid line within the box denotes the median, and the whiskers extend from the minimum to the maximum values. The dotted diamond represents the 95% confidence interval around the mean, with the dotted line indicating the mean value. FeO_2_: fraction of expired oxygen, FGF: fresh gas flow

The mean (SD) time taken to reach a FeO_2_ of 85% between the optimal and excess FGF groups was also statistically indistinguishable, i.e., 160.16 ± 67.15 versus 147.81 ± 55.5 seconds in the optimal and excess group, respectively; p-value = 0.335. The difference in the mean time required in the optimal FGF was 12.35 seconds, which accounted for +8.36%, lower than the inferiority margin of 20% taken in this study. The box-and-whisker plot shows the data dispersion and central tendencies for the time needed to achieve FeO_2_ 85% (Figure 3).

There was a minimal yet statistically significant increase in FeO_2_ from three minutes to five minutes of ventilation in the optimal FGF group; however, the increase in the excess FGF group was statistically insignificant; data are presented in Table 2.

**Table 2:** Comparison of FeO₂ values at three and five minutes between groups, analyzed using the Wilcoxon-Mann-Whitney U test ($). A two-tailed p-value <0.05 was considered statistically significant. Comparison of FeO_2_ at three minutes and five minutes within both the optimal FGF group and the excess FGF group demonstrated a statistically significant difference (paired t-test, p < 0.0001). FeO_2_: fraction of expired oxygen, FGF: fresh gas flow

Parameters	Optimal FGF (n = 50)	Excess FGF (n = 50)	W value	p-value
FeO_2_ reached (%) (three minutes)	84.52 ± 3.38	84.94 ± 3.73	W = 1127.0	0.368^$^
FeO_2_ reached (%) (five minutes)	86.02 ± 3.12	85.98 ± 3.57	W = 1199.0	0.615^$^

Thirty-one (62%) of the patients reached an FeO_2_ of 85% at three minutes in optimal FGF, while the number of patients who achieved the target FeO_2_ of 85% in three minutes in excess FGF was 40 (80%); p = 0.047. The number for the same in five minutes was 49 (98%) for both groups. Only three (6%) of the optimal and two (4%) of the excess FGF reached FeO_2_ 90% at three minutes, p = 1.00; whereas the same number were five (10%) versus two (4%), p = 0.436.

## Discussion

Our present findings question the usual current understandings that increasing FGF will shorten the time constant and improve the preoxygenation within a time frame, and can help us in reducing oxygen use during pre-intubation oxygenation, which can even contribute towards the current practice of oxygen stewardship. The present study evaluated the efficacy of achieving the preoxygenation target towards FeO_2_ of more than 85% among the excess and optimal FGF groups and did not find any notable difference in either group. We found no significant differences when comparing the mean time to reach FeO_2_ 85% between the two groups. The study also compared the mean FeO_2_ at three and five minutes and found no significant variation in the groups. Our findings show that the mean FeO_2_ in the optimal FGF group at three minutes and five minutes was 84.52% and 86.02%, respectively. In the excess FGF group, it was 84.94% and 85.98%, respectively. Therefore, our hypothesis, that providing optimal FGF, i.e., equal to MV+500mL margin, is equally effective as excess FGF, i.e., 150% of predicted MV, in achieving denitrogenation, is proved.

A randomized study, allocating 30 patients per group, analyzed the effect of three different FGF rates, 8, 10, and 12 L/min, on the efficacy of preoxygenation [16]. The authors studied the time required to reach FeO_2_ >90% using the TVB technique using BMV and found that increasing FGF reduced the time to achieve the preoxygenation target [16]. Russell et al. also studied the effect of three different FGF rates, each on 20 patients presenting for elective caesarean section, who were maintained on TVB through a standard circle system for three minutes [17]. They analyzed the effect of pre-oxygenated oxygen flow rates of 5 L/min, 10 L/min, and 15 L/min allocated randomly. The author studied the SD of fractional end-tidal oxygen at the end of three minutes and found that higher FeO_2_ values were obtained with oxygen flow rates of 10 L/min and 15 L/min compared with 5 L/min. However, pre-oxygenation did not improve with an increase in oxygen flow rate from 10 L/min to 15 L/min [17]. Although Russell et al.’s findings differ slightly from ours, when analyzed in the context of the MV, they resemble our results and hypothesis. The patients in the 5 L/min group are likely to have an FGF lower than the MV, whereas the 10 and 15 L/min groups are likely to have an FGF higher than the MV. Improving preoxygenation with the increasing FGF from 5 to 10 or 15, but failing to find so when comparing 10 with 15 L/min indicates that FGF should be optimal (equal or marginally higher than MV), but not significantly higher than MV (excess group as in our study). To prevent rebreathing, FGF generally needs to be more than MV. A fundamental approach to induction is to set the FGF to be slightly more than MV. Increasing the FGF offers little to no advantage after the set and measured inspired concentrations are close, and the extra fresh gas is wasted via the scavenging system; thus, restricting FGF to optimal (just above MV) might contribute to environmentally friendly anesthesia. Thirty-one (62%) participants in the optimal group and 40 (80%) in the excess FGF group achieved 85% FeO_2_ within three minutes. Notably, the mean time difference required to reach this level was 12.35 seconds and was statistically insignificant. This difference was far lower than the inferiority margin we took (20% versus 8.36%), indicating that the optimal FGF technique is non-inferior to the excess FGF technique. Considering that the optimal FGF for preoxygenation is expected to be more economical than excess FGF, the insignificant time advantage in delayed-sequence intubation suggests an alternative technique to improve pre-oxygenation efficiency rather than increasing FGF above MV. Potential adjustments can include modifying the method, such as employing deep breathing techniques or increasing the respiratory rate. These modifications could lead to a shorter time constant and more rapid preoxygenation.

Our study also observed that achieving an FeO_2_ level of 90% during pre-intubation oxygenation was challenging, as 93 out of 100 participants had FeO_2_ levels below 90% even after five minutes of pre-intubation oxygenation. This issue occurred, despite the machine being set to deliver 100% FiO_2_, the actual FiO_2_ delivered ranged between 92% and 96%. The use of closed-circuit is also one of the reasons that leads to a decrease in actual FiO_2_. In a closed system, the inspired gas will be saturated with water vapor and increasing inhalational agents, thereby decreasing the actual FiO_2_, despite administering 100% FiO_2_ [18]. Increasing partial pressure of inhalational agents over time probably explains the trend of reversal in the numbers, i.e., more (absolute number and percentage) of participants achieved 85% FeO_2_ in the excess FGF, but a lower number and percentage achieved 90% at five minutes than optimal FGF. The impact of inhalational agents on the pre-intubation oxygenation is thus needs to be studied to find the actual impact.

Both eco-friendly anesthesia and oxygen stewardship practice are current concerns, especially for inhalational anesthesia, as these carry the risk of environmental pollution. Increasing the ecological release of excess FGF will also increase the release of volatile agents. The total FGF used to administer anesthetic gases primarily determines the proportion of the administered gases that enter the scavenging system. Therefore, a basic approach during induction by setting the FGF slightly greater than MV will reduce waste and environmental contamination by anesthetic gases, promoting economic and environmentally friendly anesthesia practice [19].

Our study has the strength of randomization and no attrition, but it also has a few limitations that need to be mentioned. Our block randomization followed fixed numbers per block. Further, we took a non-inferiority margin of 20%, which some might argue is slightly higher. Although the measured margin difference in our study was even less than 10%, the upfront calculation of sample size with a 10% non-inferiority margin might impact the sample size and might lead our study to be underpowered. Further, the practice of volatile agents during pre-intubation oxygenation might not be universal in all set-ups, making the study results limited to those where volatile agents are concurrently used. Nevertheless, despite randomization, some baseline imbalances were still noted among the cohorts. Further, due to the inherent nature of the intervention, only the participant could be blinded. However, as the intervention was machine-delivered and outcomes were objective data from the monitor, even though the performer and data collector were non-blinded, it is unlikely to have impacted the outcome parameters.

## Conclusions

Pre-intubation oxygenation using the optimal FGF (predicted MV + 500 mL margin) is as adequate as using excess FGF (150% of predicted MV) to achieve FeO_2_ levels exceeding 85% during machine-delivered PS-based ventilation. Our findings also indicate comparable efficacy for optimal and excess FGF in maintaining key respiratory gas exchange parameters in the observed setting. However, it was noted that three minutes were insufficient to achieve the target FeO_2_ 90% using a closed circuit system and concurrent volatile agents.

## References

[1] Artime CA, Parotto M, Hagberg CA (2025). Airway management in the adult. Miller’s Anesthesia, 10th Edition.

[2] Danish MA (2021). Preoxygenation and anesthesia: a detailed review. Cureus.

[3] Gibbs KW, Semler MW, Driver BE (2024). Noninvasive ventilation for preoxygenation during emergency intubation. N Engl J Med.

[4] Zhong M, Xia R, Zhou J, Zhang J, Yi X, Yang A (2024). The comparison of preoxygenation methods before endotracheal intubation: a network meta-analysis of randomized trials. Front Med (Lausanne).

[5] Levitan RM, Behringer EC, Patel A (2017). Preoxygenation. Hagberg and Benumof’s Airway Management. 4th Edition.

[6] Mort TC (2004). The incidence and risk factors for cardiac arrest during emergency tracheal intubation: a justification for incorporating the ASA guidelines in the remote location. J Clin Anesth.

[7] Apfelbaum JL, Hagberg CA, Connis RT (2022). 2022 American Society of Anesthesiologists practice guidelines for management of the difficult airway. Anesthesiology.

[8] Frerk C, Mitchell VS, McNarry AF (2015). Difficult Airway Society 2015 guidelines for management of unanticipated difficult intubation in adults. Br J Anaesth.

[9] Nimmagadda U, Salem MR, Crystal GJ (2017). Preoxygenation: physiologic basis, benefits, and potential risks. Anesth Analg.

[10] Rajendran P, Karim HM, Panda CK, Neema PK, Dey S (2023). Preintubation machine-delivered pressure support ventilation with positive end-expiratory pressure versus manual bag-mask ventilation for oxygenation in overweight and obese patients: a randomized, pilot study. Cureus.

[11] Baxter AD (1997). Low and minimal flow inhalational anaesthesia. Can J Anaesth.

[12] Hopewell S, Chan AW, Collins GS (2025). CONSORT 2025 statement: updated guideline for reporting randomized trials. Nat Med.

[13] Nagappa M, Wong J, Singh M, Wong DT, Chung F (2017). An update on the various practical applications of the STOP-Bang questionnaire in anesthesia, surgery, and perioperative medicine. Curr Opin Anaesthesiol.

[14] (2022). Sealed Envelope Ltd. Create a blocked randomisation list. https://www.sealedenvelope.com/simple-randomiser/v1/lists.

[15] (2022). Sample size calculator. Sample size estimation in clinical research: from randomized controlled trials to observational studies. https://riskcalc.org/samplesize/.

[16] Bansal P, Chaudhari MS, Khara B (2020). Increasing fresh gas flows of oxygen in a circle absorber system for preoxygenation. Is it really efficacious?. Indian J Clin Anaesth.

[17] Russell EC, Wrench I, Feast M, Mohammed F (2008). Pre-oxygenation in pregnancy: the effect of fresh gas flow rates within a circle breathing system. Anaesthesia.

[18] Parthasarathy S (2013). The closed circuit and the low flow systems. Indian J Anaesth.

[19] Feldman JM (2012). Managing fresh gas flow to reduce environmental contamination. Anesth Analg.

